# Factors associated with depression among prisoners in southern Ethiopia: a cross-sectional study

**DOI:** 10.1186/s13104-018-3745-3

**Published:** 2018-09-03

**Authors:** Asres Bedaso, Gemechu Kediro, Tebikew Yeneabat

**Affiliations:** 1 School of Nursing, College of Medicine and Health Sciences, Hawassa University, Hawassa, SNNPR Ethiopia; 2 Department of Midwifery, College of Medicine and Health Sciences, Debre Markos University, Amhara, Ethiopia

**Keywords:** Prevalence, Depression, Prisoner, PHQ-9

## Abstract

**Objective:**

To assess the prevalence of depression and associated factors among prisoners in Hawassa Central Correctional Institution, Hawassa, SNNPR, Ethiopia.

**Result:**

56.4% of study participants had significant depressive symptoms. During Multiple logistic regression, depression was significantly associated with not participating in income generating activities inside the prison [AOR = 0.531 95% CI (0.32, 0.87)], History of Chronic disease [AOR = 2.62 95% CI (1.29, 5.32)] and history of Khat chewing [AOR = 2.47, 95% CI (1.04–5.85)].

**Electronic supplementary material:**

The online version of this article (10.1186/s13104-018-3745-3) contains supplementary material, which is available to authorized users.

## Introduction

Major depressive disorder (MDD), also known as depression, expressed by at least 2 weeks of low mood that is present across most situations; it is often accompanied by low self-esteem, loss of interest in normally enjoyable activities, loss of energy, pain without clear cause [1].

Depression is a significant contributor to the global burden of disease and affects all communities across the world. The World Mental Health Survey conducted in 17 countries found that on average 1 in 20 people reported having an episode of depression [2]. World Health Organization (WHO) states that depression is the leading cause of disability as measured by years lived with disability (YLDs) and it is the fourth leading contributor to the global burden of disease. By 2020 it is projected to reach second place in ranking [3].

Risk factors of depression include a family history of a condition; major life changes, chronic health problems and substance abuse [4]. To be sentenced to prison is among the most stressful depressive events in prisoner’s life. Also the possible cause of depression in prison is, memorizing past illegal acts, the prisoners try to relieve the moments of their crime, and this makes them feel guilty and sorrow. Constantly having these thoughts may result in severe depression and prolonged stay in the prison may lead to intense depression. In addition; prisoners feel loneliness, as they are isolated from their family and loved ones. Mostly life with other prisoner and the prison environment by itself to be confined to restricted space also leads to depression [5].

The prevalence of psychiatric illness in correctional setting is significantly elevated; with higher than community rates reported for most mental disorder [6]. Epidemiological studies conducted among prisoners have shown a high prevalence of psychiatric morbidity. The magnitude of severe mental disorder was five to ten times higher among prisoners compared to general population [7].

As important as correctional facilities are for correctional purposes, the institutions could be destructive too. Local as well as international evidence firmly established that incarceration has severe negative public health consequences. Accordingly, the much intended action to promote correction could be a cause for severe physical and mental health deterioration among the incarcerated and the society at large [8].

Depression is especially prevalent in prison populations [9]. In our society the disproportional high rate of mental disorders in prisons is related to several factors starting from a misconception that all people with mental disorders are a danger to the public. The increased risk of suicide in prisons (often related to depression) is unfortunately, one common manifestation of the cumulative effect of these factors [10].

Globally depression is the second leading cause of disability, with slightly more than 4% of the world’s population diagnosed with it. More than 5% of the population suffers from depression in the Middle East, North Africa, sub-Saharan Africa, eastern Europe and the Caribbean. The most depressed country is Afghanistan, where more than 1 in 5 people suffer from the disorder. The least depressed is Japan, with a diagnosed rate of less than 2.5% [11].

The world health organization estimates that about 350 million people affected by depression, with an increase of more than 18% between 2005 and 2015. The national institute for mental health (NIMH) estimates that in the United States, 16 million adults had at least one major depressive episode (MDE) in 2012. On the other hand, the prevalence of depression in Ethiopia was reported to be 5% according to the Ethiopian federal ministry of health report of 2012, and WHO survey in collaboration with Jimma University shows that the prevalence of depression in Ethiopia was 9.1% [12]. National survey of 2014 states the pooled prevalence of depression from 8 studies in Ethiopia was 11% [13].

A systematic review of 62 surveys in 12 countries prisons involving 22,790 inmates found that, among males, 26% were violent offenders, 3.7% had psychotic illnesses, 10% suffered from major depression and 65% had a personality disorder, of which 47% had antisocial and among female prisoners 4% had a psychotic illness, 12% had a major depression and 42% had a personality disorder, of which 21% has antisocial [14].

Despite scanty evidence regarding the situation of mental health in Ethiopia, there is enough evidence to worry about and act accordingly. In a survey of federal prisons in Addis Ababa and Kaliti, 61.9% of prisoners were found to have different levels of mental distress [15].

There were no sufficient studies conducted in Ethiopia, especially in southern region to determine prevalence of depression and its associated factors among inmates in prison. Therefore this research aimed at determining the prevalence of depression and its associated factors among prisoners in Hawassa central correctional institution, SNNPR, Ethiopia.

## Main text

### Study design

Institution based cross sectional study design was employed.

### Study area and period

The study was conducted in Hawassa Central Correctional Institution from March 1–30, 2018. Hawassa Central Correctional Institution Located in Ethiopia, southern Nations Nationalities and people Regional State, Hawassa city. It is established in 1966 G.C which is one of the 23 Correctional Institutions in the region that serves as a center for other 22 prisons. It is located at western part of Hawassa at the base of Alamura Mountain. Currently this prison held 2317 populations (2220 males and 97 females). Of the total prisoners, 1640 are convicted prisoner, (1600 males and 40 females). Out of the total convicted prisoners 42 inmates are lifelong prisoner (40 male, 2 female) [16].

### Participant

All prisoners held at Hawassa Central Correctional Institution were considered as Source population. Prisoners who are available during the study period were considered as study populations. Individual prisoner in Hawassa Central Correctional Institution is the study unit. The study included all prisoners whose age is 18 years and above. Awaiting trial and critically ill (can’t give response) prisoners were excluded from the study.

Single population proportion formula (with a 5% margin of error, 95% confidence level and 43.8% [17] proportion was used to calculate sample size. Then we used correction formula since the total numbers of convicted prisoners were < 10,000. Adding 10% non-response rate, the final sample size was 335. Simple random sampling technique was applied to select study participants.

### Instrument

Depression among prisoners in the last 15 days is assessed by the Amharic version of Patient health questionnaire (PHQ9). PHQ-9 measurement ranges from zero to three. The PHQ-9 has demonstrated acceptable reliability and validated for use in Ethiopia. A PHQ-9 score ≥ 5 was considered as significant for meeting the symptoms of depression. The PHQ-9 incorporates the DSM IV depression criteria along with other leading depression symptoms into a brief self-report tool [18]. Semi structured questionnaires were used to assess substance use, socio-demographic and other clinical factors.

Data were collected by using Interviewer administered technique. Data were compiled, entered and analyzed by using SPSS version 20. Binary and multiple logistic regression models were used to identify factors associated with depression. Adjusted odds ratio with 95% confidence interval and *P* value of 0.05 was used to determine the final model.

### Results

#### Socio demographic characteristics

Three hundred thirty-five respondents were participated in the study making a response rate of 100%. The median age of the study participants was 27 years (SD ± 7.53). The majority of the respondents were male (97.6%), 44.5% were orthodox religion followers and 61.2% were single in marital status (Table 1).

**Table 1:** Socio demographic characteristics of prisoners in Hawassa central correctional institution, SNNPR, Ethiopia, 2018 (n = 335)

Covariate	Frequency (%)
Age	
18–25	142 (42.4%)
26–33	119 (35.5%)
34–41	52 (15.5%)
≥ 42	22 (6.5%)
Sex	
Male	327 (97.6%)
Female	8 (2.4%)
Religion	
Orthodox	149 (44.5%)
Protestant	128 (38.2%)
Muslim	37 (11.0%)
Catholic	12 (3.6%)
Others	9 (2.7%)
Marital status	
Single	205 (61.2%)
Married	112 (33.4%)
Divorced	18 (5.4%)
Ethnic group	
Sidama	142 (42.4%)
Wolaita	83 (24.8%)
Oromo	48 (14.3%)
Amhara	43 (12.8%)
Others	19 (5.7%)
Educational level	
Illiterate	100 (29.8%)
Primary education (grades 1–8)	84 (25.1%)
High school (grade 9–12)	93 (27.8%)
College and above	58 (17.3%)
Participating in income generating activities in prison	
Yes	98 (29.3%)
No	237 (70.7%)
Duration of imprisonment (years)	
≤ 1	125 (37.3%)
1–5	135 (40.3%)
≥ 5	75 (22.4%)

#### Clinical characteristics of prisoners

From this study participants, 43 (12.8%) of the prisoners had family history of psychiatric illness, 17 (5.1%) of prisoners were cardiac patients (Table 2 and Additional file 1).

**Table 2:** Clinical characteristics of prisoners in Hawassa central correctional Institution, SNNPR, Ethiopia, 2018 (n = 335)

Covariate	Frequency (%)
Family history of psychiatric illness	
Yes	43 (12.8%)
No	292 (87.2%)
Prisoners history of chronic diseases	
Yes	30 (9.0%)
No	305 (91.0%)
Suicidal thought	
Yes	221 (66.0%)
No	114 (34.0%)

#### Prevalence of depression

According to PHQ-9, 189 (56.4%) of study subjects were identified as having a depressive episode in the 2 weeks preceding the study. From total of study participant, One hundred sixty (34.6%) scored for mild depression, 52 (15.5%) for moderate and 21 (6.3%) for severe depression (Additional file 2).

#### Substance abuse

From the total study participants, 10.1% of prisoners had history of cigarette smoking while 14.3% of prisoner had history of chewing Khat and 11.9% had history of alcohol drinking.

#### Factors associated with depression

Variables that had significant association on Binary logistic regression analysis at P-value < 0.2 were entered for multiple logistic regression analysis to test for its significance. During multiple logistic regression, participating in income generating activities (having occupation) in the prison, history of chronic disease, and history of chat chewing are significantly associated with depression (Table 3).

**Table 3:** Factors associated with depression (bivariate and multivariate logistic regression) among prisoners in Hawassa central correctional institution, SNNPR, Ethiopia (n = 335)

Explanatory variables	Depression		COR (95% CI)	AOR (95% CI)	P-value
	Yes	No			
Age					
18–25	81	61	1		
26–33	70	49	5.311 (0.579, 48.727)		
34–41	28	24	5.714 (0.620, 52.693)		
42–49	9	8	4.667 (0.488, 44.637)		
50–57	1	4	4.500 (0.413, 49.077)		
Educational status					
Illiterate	55	45	0.694 (0.357, 1.349)		
Primary education	51	33	0.877 (0.439, 1.752)		
High school	46	47	0.555 (0.284, 1.088)		
College and above	37	21	1		
Participating in income generating activity inside prison					
Yes	44	54	1		
No	145	92	0.517 (0.321, 0.832)	0.531 (0.322, 0.877)	0.013*
Past history of cigarette smoking					
Yes	24	10	1.978 (0.914, 4.280)		
No	165	136	1		
Past history of Khat chewing					
Yes	36	12	2.627 (1.314, 5.255)	2.478 (1.049, 5.857)	0.039*
No	153	134	1		
Chronic disease^a^					
Yes	39	12	2.903 (1.460, 5.775)	2.621 (1.291, 5.323)	0.008*
No	150	134	1		
Duration of imprisonment (years)					
< 1	69	56	1		
1–5	71	64	0.654 (0.362, 1.182)		
> 5	49	26	0.589 (0.328, 1.055)		

* P-value ≤ 0.05 (significant association)

^a^Any of diseases like cardiac, HTN, DM, HIVAIDS, liver or kidney diseases

### Discussion

As to the researchers knowledge this is the first study conducted in southern region prisons related to prevalence of depression and associated factors. The overall prevalence of depression among prisoners is 56.4%. This result was higher than the study conducted in Norwegian prison (46%) [19], United States prison which ranges from 9 to 29% [20], Iran (42%) [21], eastern Nepal prisons (35.5%) [22] (30.6%) [23], in Cameroon among medical students, Nigerian maximum security prison (42%) [24] and North West Amhara prison (43.8%) [17]. Economic status of study area, civilization difference, place of the study and tool difference might be the possible causes for discrepancy in prevalence of depression.

On the other hand, this study revealed that low prevalence of depression than another studies conducted in Indian Rajahmundry central jail (81.18%) [25] and Nigeria medium security prison in Benin city (72.6%) [26]. The possible reason for the difference might be due to different socio-demographic characteristics of study participants, time of the study and tool differences.

The prevalence of depression in the current study is much higher compared with general population in Ethiopia (9.1%) [12] as well as the British national reports [9]. The possible reason of depression prevalence to be high in prison might be due to stressful environment of the prison, isolation from family, lack of freedom of movement in prison compared to outside to prison population.

Prisoners who had chronic disease were about three times more prone to develop depression than those who are healthy (AOR = 2.62 95% CI 1.291–5.323, P = 0.008). This might be due to stresses related to chronic diseases is a condition that lasts for long time and thoughts the disease might not be cured. This finding is similar to results from a study conducted in eastern Nepal, depression was likely in prisoners who had current health problem (OR = 1.75, 95% CI = 1.16–2.64, P = 0.007) [22], also this finding is comparable to the results from a study conducted in Norwegian prison [27] and study conducted among general population of Ethiopia (OR = 4.2, 95% CI 3.18–5.57, P < 0.0001) [12].

This study also shows that prisoners who were participating in income generating activities were 47% less likely to develop depression (AOR = 0.531 95% CI 0.322–0.877, P = 0.013) than the counter parts. Work engagement positively improved with quality of life of individuals (i.e., improved individual and family satisfaction) and productivity, which will keep an individual mentally health. In contrary, study conducted among women in rural Ethiopia founds that employment were not associated with depression [28]. This possible explanation to this difference might be socio-demographic characteristics of study participant.

Prisoners who chew Khat prior to incarceration were about two times more likely to develop depression (AOR = 2.47 95% CI 1.049–5.85, P = 0.039) than those who did not chew Khat. This implies people who chew khat for relaxant and euphoric effects for a moment and to reduce stress, but after withdrawal this may worsen the stress and in the long term lead to depression. This is different from study conducted among Somalia immigrants [29]. This possible explanation to this difference might be socio-demographic characteristics of study participant.

### Conclusion

This finding revealed that there is high prevalence of Depression in Hawassa central correctional institution prisoners (56.4%). In addition; chronic disease status, not participating income generating activity inside the prison and Khat chewing prior to incarceration were significantly associated depression.

This study suggests that the institution need to provide proper psychiatric service to diagnose and treat prisoners with depression. The institution need to facilitate income generating activities inside prison, so as to relief negative feelings. It will be better if researchers conduct interventional research in order to show the way to reduce the prevalence of depression through interventional strategies.

## Limitation

Since the study design is cross-sectional study, it does not allow inferring the causation. Also there might be possibility of omitted variables bias.

## Additional files

**Additional file 1.** Chronic Medical Illness among Prisoner in Hawassa Central Correctional Institution, SNNPR, 2018.

**Additional file 2.** Severity of depression among prisoners in Hawassa Central Correctional Institution, SNNPR, Ethiopia, 2018 (n = 335).

## Abbreviations

AOR: adjusted odds ratio
CI: confidence interval
DSM IV: Diagnostic and Statistical Manual Version IV
G.C: gregorian calendar
MDD: major depressive disorder
MDE: major depressive episode
PHQ: Patient Health Questionnaire
SD: standard deviation
SPSS: Statistical Package for Social Science
YLDs: years lived with disability

## References

[1] National Institute of Mental Health, Depression, Transforming the understanding and treatment of mental illness. 2016. https://www.nimh.nih.gov/health/topics/depression/index.shtml.

[2] World Federation for Mental Health, Depression. A global crises book. 2012. p 6. http://www.who.int/mental_health/…/depression/wfmh_paper_depression_wmhd_2012.pdf.

[3] World Health Organization. Depression fact sheets, updated February 2017. http://www.who.int/gho/mortalityburdendisease/en/index.html.

[4] American Psychiatric Association. Diagnostic and statistical manual of mental disorder.

[5] Thomas L. Prisoner depression and low mood, newly medical life science. 2017 https://www.news-medical.net/health/Prisoner-Depression-and-Low-Mood.aspx.

[6] Johann B (2005). Epidemiology of mental illness in a correctional system. Curr Opin Psychiatry.

[7] Falissard BLJ, Gasquent I, de Duburc A (2006). Prevalence of mental disorder in French prison for men. BMC Psychiatry..

[8] Seyum D. Growing incarceration and the public health dilemma in Ethiopia, the reporter magazine, Addis Ababa. 2017. https://archive.thereporterethiopia.com/sites/default/files/Pdf%20Archive/Reporter-Issue-1116.pdf.

[9] Brugah T (2005). Psychosis in the community and prisoners: a report from a British national survey of psychiatric morbidity. Am J Psychiatry.

[10] Federal Democratic Republic of Ethiopia Ministry of Health, National Mental health strategy 20102/13-2015/16. 2012. http://www.mhinnovation.net/sites/default/files/downloads/innovation/reports/Ethiopia-national-mental-health-strategy-2012-1.pdf.

[11] Dewey C. A stunning map of depression rates around the world, the Washington post magazine. 2013. https://lbfromlv.wordpress.com/2013/11/08/a-stunning-map-of-depression-rates-around-the-world-by-caitlin-dewey-november-7-at-700-am.

[12] Hailemariam et al. Prevalence of depression and associated factors: finding from national health survey. Int J Men Health Syst. 2012, p. 4. http://www.ijmhs.com/content/6/1/23. 10.1186/1752-4458-6-23 PMC3511231 23098320

[13] Teresa B (2014). Prevalence of depression and potential risk factors in Ethiopia. Ethiop J Health Sci..

[14] Fazel S, Danesh J (2002). Serious mental disorder: a systemic review of 62 surveys. Lancet.

[15] Asgedom A (2008). Prevalence of mental distress among federal prisoners in Ethiopia in department of psychiatry.

[16] Annual Plan. Hawassa Central correctional Center. 2010.

[17] Beyen (2017). More than eight in nineteen inmates were living with depression at prison of northwest Amhara regional state, Ethiopia, a cross sectional study design. BMC Psychiatry.

[18] Roy W (2016). World prison population list. World Prison Brief..

[19] Værøy H (2011). Depression, anxiety, and history of substance abuse among Norwegian inmates in preventive detention: reason to worry?. BMC Psychiatry..

[20] Seth J (2015). Prins, the prevalence of mental illnesses in U.S prisons: systematic review. PMC. Psychiatry.

[21] Valizadeh R, Veisani Y, Delpisheh A, Kikhavani S, Sohrabnejad A (2017). Major depression and psychiatric disorders in Iranian prisoners based on a clinical interview: a systematic review and meta-analysis. Shiraz E Med J..

[22] Shrestha (2017). Depression among inmates in regional prison of eastern Nepal. BMC Psychiatry.

[23] Ngasa (2017). Prevalence and factors associated with depression among medical students in Cameroon. BMC Psychiatry.

[24] Nwaopara, Stanley (2015). Prevalence of depression in Port Harcourt prison. J Psychiatry.

[25] Data PV (2015). Prevalence of depression and assessment of its severity among prisoners of central prison, Rajahmundry, India. Indo Am J Pharm Res..

[26] Osasona SO, Koleoso ON (2015). Prevalence and correlates of depression and anxiety disorder in a sample of inmates in a Nigerian prison. Int J Psychiatry Med.

[27] Iversion VC (2014). Psychological distress perceived health in inmates in Norwegian Prisons. Scand J Public Health..

[28] Deyesa N (2008). Depression among women in rural Ethiopia as related to socioeconomic factor: a community based study on women in reproductive age groups. Scand J Public Health.

[29] Kamal B, Nasir W (2010). Trauma, khat and common psychotic symptoms among Somalia immigrants. J Ethno Pharmacol.

