# Supplementary material for: Factors associated with depression among prisoners in southern Ethiopia: a cross-sectional study
Source: BMC Res Notes. 2018 Sep 3;11:637. doi: 10.1186/s13104-018-3745-3 (PMC6122681; doi:10.1186/s13104-018-3745-3)
Supplement: Supplementary file 1 — Additional file 1. Chronic Medical Illness among Prisoner in Hawassa Central Correctional Institution, SNNPR, 2018. [file 13104_2018_3745_MOESM1_ESM.docx]

Additional File 1: Chronic Medical Illness among Prisoner in Hawassa Central Correctional Institution, SNNPR, 2018
