# Supplementary material for: Factors associated with depression among prisoners in southern Ethiopia: a cross-sectional study
Source: BMC Res Notes. 2018 Sep 3;11:637. doi: 10.1186/s13104-018-3745-3 (PMC6122681; doi:10.1186/s13104-018-3745-3)
Supplement: Supplementary file 2 — Additional file 2. Severity of depression among prisoners in Hawassa Central Correctional Institution, SNNPR, Ethiopia, 2018 (n = 335). [file 13104_2018_3745_MOESM2_ESM.docx]

Additional File 2: Severity of depression among prisoners in Hawassa Central Correctional Institution, SNNPR, Ethiopia, 2018 (n=335).
